# Factors Associated with the Social Behaviour of People with Alzheimer’s Dementia: A Video Observation Study

**DOI:** 10.3390/brainsci15111205

**Published:** 2025-11-08

**Authors:** Jasmine Shaw, Fern Rodgers, Deniz Eda Kavustu, Yuding Wang, Sarah Assaad, Gill Livingston, Andrew Sommerlad

**Affiliations:** 1 Division of Psychiatry, University College London, Maple House, 149 Tottenham Court Road, London W1T 7NF, UK; jasmine.shaw.23@alumni.ucl.ac.uk (J.S.); fern.rodgers.24@ucl.ac.uk (F.R.);; 2 North London NHS Foundation Trust, St Pancras Hospital, London NW1 0PE, UK

**Keywords:** Alzheimer’s dementia, social behaviour, social functioning, social cognition

## Abstract

**Background/Objectives:** People with Alzheimer’s dementia (AD) experience distressing changes in social behaviour. However, little is understood about whether social behaviour is associated with support provided by, or familiarity with, conversation partners. We aimed to explore the association between support provided by, and familiarity with, conversation partners and the social behaviour of people with mild AD during conversation. **Method:** We designed an exploratory within-subjects study wherein conversations between 19 participants with mild AD and a familiar informant, followed by an unfamiliar researcher, were video-recorded and double-rated using two measures of social behaviour (Social Observation Inventory and Measure of Participation in Conversation—Dementia), and one measure of support from the conversation partner (Measure of Support in Conversation—Dementia). Multilevel linear regression with within-subject clusters was used to explore adjusted associations between support and familiarity and social behaviour. **Results:** Greater support in conversation was associated with more appropriate participation in social conversation of participants with AD. In fully adjusted models, every 1-point increase in MSC-D score was associated with a 0.29 (95% CI: 0.14 to 0.44) increase in MPC-D score and a 1.59 (95% CI: 0.87 to 2.32) increase in SOI score. Familiarity with the conversation partner was not associated with the social behaviour of the participants with AD. **Conclusions:** We found evidence for an association between social behaviour in AD and support provided by unimpaired conversation partners, but the numbers were small, and this should be interpreted cautiously. Future research should continue this hypothetical lead to expand our understanding of how support and familiarity influence social behaviour to inform potential interventions.

## 1. Introduction

Dementia commonly results in progressive cognitive and social functioning decline [1], leading to changes in social behaviour that may include reduced social tactfulness, a lack of manners, interpersonal boundary infringements, and reduced use of communicative gestures [2]. This can be observed during the prodromal and mild stages of AD [3], can affect social relationships and interactions, and is distressing for people with dementia and their family caregivers [4]. Social behavioural changes in behavioural variant frontotemporal dementia (bvFTD) may be consequences of impaired social perception and other social cognitive processes [5] and impaired social knowledge and control [6], leading to negative changes in social behaviour that would otherwise be informed by context-specific cues. This indicates that, as well as considering the contribution of social cognitive impairment on social behaviour in dementia, there is a need to consider other contextual factors as potentially important influences.

It is often recommended that caregivers of people with dementia practice supportive communication during social interactions and conversations to combat associated distress [7]. One review found that the conversational behaviour from nonimpaired conversation partners influenced the behaviours of people with dementia [8], for example, by providing scaffolding to include and sustain the involvement of the person with dementia in the conversation. For people with bvFTD, the influence of these supportive behaviours is thought to be underpinned by interactional and transactional functions of the conversation that have also been associated with greater participation in conversation for people with aphasia and traumatic brain injury [9,10,11]. However, there is little research into the association between support from conversation partners and social behaviour in AD and an absence of effective interventions improving social cognition [12].

Our preliminary patient and public involvement work with family caregivers of individuals with AD further suggested that social behaviours may differ depending on the familiarity of the conversation partner. Principles of cognitive rehabilitation suggest that familiar contexts may reduce cognitive load, improving the performance of specific tasks [13], and there is evidence that reducing cognitive load can promote general cognitive functioning in cases of AD [14]. Other supporting evidence comes from a study of autobiographical memory, suggesting that familiarity may have supporting effects on the retrieval of social information for people with AD [15], and a destination memory framework, which poses familiarity as a measurable feature in its association with social cognition during social interaction [16]. The effects of involving and training family caregivers of people with dementia in care plan models are positive in practice [17], aligning with the idea that familiarity with a conversation partner may positively influence the social behaviour of people with AD. However, this has not, to our knowledge, been empirically studied. An understanding of the effect of familiarity with, and the level of support provided by, conversation partners of people with dementia may inform future approaches to improving communication through family caregiver training interventions.

We therefore aimed to observe the social behaviour of people with mild AD and explore associations with the support provided by, and familiarity with, unimpaired conversation partners, addressing the following research questions:

1. Is support from the conversation partner associated with the social behaviour of people with AD?
2. Is familiarity with the conversation partner associated with the social behaviour of people with AD?

We hypothesised that greater support provided by, and familiarity with, unimpaired conversation partners would be associated with more appropriate participation in social conversation from people with mild AD.

## 2. Materials and Methods

### 2.1. Study Design

This was a within-subjects (repeated measures) exploratory study of conversations between people with AD and familiar and unfamiliar conversation partners. This study received ethical approval from the Wales 6 National Health Service Research Ethics Committee (23/WA/0157).

### 2.2. Participants

We recruited 19 participants with mild AD and 19 of their relatives as familiar conversation partners (n = 19 dyads). Two researchers (FR, AS) acted as unfamiliar conversation partners for the participants with AD. There were therefore 38 datapoints.

- The participants with AD were diagnosed with probable AD from UK National Health Service diagnostic memory clinics (using ICD-10 criteria) [18]. They were required to have scored ≥20 within the previous 6 months on the mini-mental state examination [19] or equivalent score [20] on another test, such as the Addenbrooke’s Cognitive Examination version 3 (ACE-III) [21], indicating mild dementia. The participants were required to be aged 50 years or older and speak conversational English.
- The familiar conversation partners were either the son (2; 11%), daughter (4; 21%), spouse (11; 57%), or friend (2; 11%) of the participants with AD and needed to have at least monthly contact with them to ensure familiarity. They were required to speak conversational English and be aged ≥18 years.
- The two researchers who acted as unfamiliar conversation partners did not have regular contact with the other participants prior to this study and were not involved in rating the video-recorded conversations.

No further exclusion criteria were applied.

### 2.3. Procedures

Dyads of participants with AD and their relatives were provided with information about this study by their clinicians at the Camden and Islington NHS Foundation Trust and the North East London NHS Trust and invited to participate. Interested dyads met with the study team at university premises (10; 53%) or at the participant with AD’s home (9; 47%), according to the dyad participants’ preferences. All the participants provided informed written consent and demographic information, and the participants with AD completed the ACE-III [21]. A further four questionnaires for the participants with AD and six questionnaires for their relatives were completed, lasting around 45–60 min, but were not included in the current study.

We then collected video-recordings of 38 conversations; the participants with AD had two conversations each: one with their familiar relative and one with an unfamiliar researcher. First, two GoPro cameras on tripods were set up behind the dyad participants in conversation to capture their viewpoint of the individual with whom they were conversing. Using a standard script, the dyads were asked to have a conversation lasting up to 10 min about the person with AD’s favourite food, which was chosen as an emotionally neutral topic to offer standardisation of content across all recordings. The relatives (familiar conversation partners) were provided a prompt sheet with four suggested questions for conversation continuity (see the Appendix A), and both participants in the conversation were encouraged to contribute to the discussion. After this conversation ended (either naturally between 5 and 10 min or when 10 min was reached), the recordings were stopped. A researcher (unfamiliar conversation partner) replaced the relatives in the room, sitting across from the participant with AD. The recordings were re-started, and the person with AD completed a picture description task [22] with the researcher before they had a 5 to 10 min conversation about the same neutral topic; the researcher also used the prompt sheet for conversation continuity. Data were collected between August 2023 and October 2024. The participant dyads were given a GBP 20 gift voucher as compensation.

### 2.4. Data and Measurement

Three researchers independently rated the 38 video-recorded conversations; DK observed 28, YW observed 10, and JS double-rated all 38. Each conversation was viewed from the perspective of the participant with AD, during which their unimpaired conversation partner’s supportive behaviours were coded using the Measure of Support in Conversation—Dementia (MSC-D) [11], and from the perspective of the unimpaired conversation partner, during which the participant with AD’s social behaviour was coded using the Measure of Participation in Conversation—Dementia (MPC-D) [11] and the Social Observation Inventory (SOI) [23]. See Mok and colleagues’ Supplementary Material for the MSC-D and MPC-D manuals and Mendez and colleagues’ report for the SOI items.

The MSC-D was designed to quantify the support provided by the conversation partner of a person with dementia according to four functions of conversation:

- Acknowledging competence, which assesses the appropriateness of the flow of conversation and the conversation partner’s sensitivity and responsiveness to the person with dementia;
- Revealing competence (understanding), which assesses whether the conversation partner ensures the person with dementia understands topics and questions;
- Revealing competence (responding), which assesses whether the conversation partner ensures the person with dementia is able to get their message across;
- Revealing competence (verification), which assesses whether the conversation partner ensures that they have correctly received the message from the person with dementia.

These functions were scored on a Likert scale ranging from 0 to 4, with 0.5 increments (0 = very poor support; 4 = very good support). Higher scores indicated better support in conversation, with a potential total score ranging from 0 to 16. Previous use of this measure indicated excellent inter-rater reliability across all functions (ICC = 0.76 to 0.83) except revealing competence anchors for verification, which had good reliability (ICC = 0.72) [11].

The MPC-D, developed alongside the MSC-D, quantifies the social behaviour of people with dementia according to two domains of participation in conversation:

- Interaction, which assesses whether the person with dementia is socially appropriate in their verbal, vocal, and nonverbal communication, engages, and maintains interaction;
- Transaction, which assesses whether the person with dementia conveys information and content by providing information, opinions, and feelings appropriate to the context.

These domains were scored on a Likert scale ranging from 0 to 4, with 0.5 increments (0 = no participation; 4 = full participation). Higher scores indicated more appropriate social behaviour, with potential total scores ranging from 0 to 8. Previous use of this measure indicated excellent inter-rater reliability across both functions (ICC = 0.90 to 0.95) [11].

The SOI quantifies the appropriateness of social behaviour of people with dementia according to ten verbal and ten nonverbal behaviours rated using 6-point Likert scales ranging from 0 to 5 each (0 = never; 5 = very frequent). Higher scores indicate more appropriate social behaviour, with potential total scores ranging from 0 to 100 (50 for verbal and 50 for nonverbal behaviours). Previous use of this measure suggests excellent inter-rater reliability, with scores of 0.90 or higher for all 20 items, and discriminant validity because participants with bvFTD scored, as expected, lower than those with AD [23].

Covariates of the participants with AD’s age and ACE-III score were used to adjust for associations of age-related cognitive decline and cognitive impairment, respectively. The ACE-III is a screening tool, validated for use with AD patients, that measures cognitive impairment based on task performance across five domains: attention, memory, fluency, language, and visuospatial function [21]. Scores range from 0 to 100, with lower scores indicating greater cognitive impairment. The covariate of conversation location (participant’s home or UCL research department) was also adjusted for, based on research suggesting the potential for familiar associations and context to influence social behaviour [5,24].

The data were organised so that the number of observations reflected the number of conversations (n = 38). Hence, the data contained 19 within-subject clusters of participants with AD who had two conversations each.

### 2.5. Analyses

The scores from the two raters for each conversation were combined using mean scores for each item on all three measures [25]. We assessed the inter-rater reliability of the MSC-D, MPC-D, and SOI scores using intraclass correlation coefficient (ICC) two-way absolute agreement analyses of average measures [26,27]. ICC scores were performed using the data from all conversations (n = 38) to satisfy ICC power calculations [28].

We then used Pearson’s correlation coefficients to explore associations between the total MSC-D scores and the total MPC-D and SOI scores. Multilevel linear regression analyses were used to explore the association between MSC-D scores and MPC-D and SOI scores in two separate models accounting for the 19 within-subject clusters [29]. We used random intercepts only to reduce overfitting of data [30] and adjusted these models sequentially for the covariates of age, ACE-III score, conversation location, and conversation partner. We then repeated the multilevel linear regression models to examine the associations between the four individual MSC-D domains (acknowledging competence, revealing competence for verification, revealing competence for responding, and revealing competence for understanding) and MPC-D and SOI scores in eight separate models to explore separate contributions of supportive functions to the social behaviour of the participants with AD. Overall, we used 10 fully adjusted models (two main models and eight for further exploration of support functions) to address our first research question.

We used Wilcoxon rank-sum tests, with exact *p* values, to explore differences in the MSC-D, MPC-D and SOI scores between the conversations with familiar and unfamiliar conversation partners. Multilevel linear regression (accounting for the 19 within-subject clusters) was used to explore the association between the conversation partner (familiar or unfamiliar) and MPC-D and SOI scores in two separate models. We used random intercepts only and adjusted these models sequentially for the covariates of age, ACE-III scores, conversation location, and MSC-D scores. Overall, we used two main fully adjusted models to test our second research question.

We tested assumptions for multilevel linear regression models and conducted sensitivity analyses where the assumptions for these models were not met (see the Appendix A). For the sensitivity analyses, we conducted bootstrapping of reported coefficients for the four main fully adjusted models addressing the research questions and also repeated the four main models, adjusting for explanatory variables and covariates with potentially curvilinear relationships with the outcome by introducing their quadratic terms and adjusting for potential heteroscedasticity of residuals by specifying robust standard errors. A power calculation showed that our sample size of 19 conversations provided 80% power at a significance level of 5% to find an effect size of 0.56.

Statistical analyses were conducted using Stata v18.

## 3. Results

### 3.1. Descriptive Statistics

The participants included people with AD, with 19 family or friend informants and 2 researchers as conversation partners (see demographic and clinical characteristics in Table 1). The participants with AD ranged in age from 70 to 92 years, and their family or friend informants’ ages ranged from 40 to 86 years. The participants with AD had ACE-III scores between 50 and 85. The majority of the participants were White and female, and English was commonly their first language. The two researcher participants (i.e., the non-familiar conversation partners) were a 29-year-old White woman and a 40-year-old White man who both had postgraduate degree-level education and spoke English as their first language. The video durations were between 5 and 11 min.

ICC analyses of inter-rater reliability for the current study were conducted between double-rated scores on the MSC-D, MPC-D, and SOI (see Table 2). According to Koo and Li’s [27] guidelines, we established excellent inter-rater reliability for total MSC-D ratings (ICC = 0.90) and good reliability across all domains of the MSC-D (ICC = 0.75 to 0.87), except revealing competence anchors for verification, which had moderate reliability (ICC = 0.64).

We found good inter-rater reliability for the total MPC-D ratings (ICC = 0.86) and excellent inter-rater reliability for the total SOI ratings (ICC = 0.92). The scores were then combined to find the mean scores for further statistical analyses.

### 3.2. Is Support from the Conversation Partner Associated with the Social Behaviour of People with AD?

Pearson’s correlation coefficients showed a strong positive correlation between scores on the MSC-D and the MPC-D (*r* = 0.55; *p* < 0.001) and SOI (*r* = 0.53; *p* = 0.001).

Multilevel linear regression analyses were conducted to assess the association between MSC-D scores and MPC-D and SOI scores (see Table 3). In model 1, every 1-point increase in MSC-D score was associated with a 0.27 (95% CI: 0.16 to 0.39) increase in MPC-D score. After adjusting for covariates of age, ACE-III scores, conversation location, and conversation partner, every 1-point increase in MSC-D score was associated with a 0.29 (95% CI: 0.14 to 0.44) increase in MPC-D score.

In model 2, every 1-point increase in MSC-D score was associated with a 1.33 (95% CI: 0.78 to 1.88) increase in SOI score. After adjusting for covariates, a 1-point increase in MSC-D score was associated with a 1.59 (95% CI: 0.87 to 2.32) increase in SOI score.

Multilevel linear regression analyses were conducted to assess the association between individual MSC-D domain scores and MPC-D and SOI scores (see Table 4). In the fully adjusted models, there was no association between Acknowledging Competence score and either MPC-D score or SOI score. However, every 1-point increase in Revealing Competence for Understanding score was associated with a 1.10 (95% CI: 0.48 to 1.73) increase in MPC-D score and a 7.33 (95% CI: 4.66 to 10.05) increase in SOI score; every 1-point increase in Revealing Competence for Responding score was associated with a 0.79 (95% CI: 0.33 to 1.25) increase in MPC-D score and 4.94 (95% CI: 2.86 to 7.03) increase in SOI score; and every 1-point increase in Revealing Competence for Verification score was associated with a 0.57 (95% CI: 0.17 to 0.96) increase in MPC-D score and 2.78 (95% CI: 0.67 to 4.89) increase in SOI score.

### 3.3. Is Familiarity with the Conversation Partner Associated with the Social Behaviour of People with AD?

The two-sample Wilcoxon rank-sum tests showed no differences between scores on the MPC-D or SOI between the conversations with relatives and researchers, but the researchers scored higher on the MSC-D (median = 14.0; inter-quartile range [IQR] = 1.0) than the relatives (median = 12.0; IQR = 3.5; z = −3.46, *p* < 0.05).

Multilinear regression analyses were conducted to assess the association between the conversation partner and MPC-D and SOI scores (see Table 5). In model 3, the MPC-D scores were 0.61 (95% CI: 0.06 to 1.15) higher during the conversations with unfamiliar compared to familiar conversation partners, but after adjusting for MSC-D scores, evidence for this relationship ceased.

In model 4, there was no evidence for a relationship between the conversation partner and SOI score in either the unadjusted or fully adjusted model.

## 4. Discussion

To our knowledge, this was the first quantitative study to explore an association between support from, and familiarity with, conversation partners and the social behaviour of people with AD. We found preliminary evidence that greater support from a conversation partner was associated with more appropriate social behaviour of the participants with AD, even after adjusting for covariates of age, ACE-III score, conversation location, and conversation partner and in sensitivity analyses. However, we found that the appropriateness of the social behaviour and participation of the participants with AD in conversation did not differ depending on the familiarity of the unimpaired conversation partner in adjusted analyses.

The evidence we found for an association between provided support and social behaviour in AD is supported by a review of conversational analytic studies, suggesting that conversational strategies provide support for people with dementia to exchange information and engage in social interaction [8], and qualitative data linking adaptation of carers to social behavioural difficulties of their relative with dementia to improved social functioning [4]. Further, an observational study suggested that carer strategies can affect other behavioural domains of people with dementia over a 1-year follow-up [31]. Our study adds to this literature through direct observation and independent rating of behaviour. The cross-sectional nature of our study means that it is not possible to prove direction of causation—for example, it is possible that better social behaviour may facilitate more support from the conversation partner—but reverse causality is less likely since the effect of support on social behaviour has been observed across different clinical populations (e.g., [9]). Therefore, our findings promote the notion that wider social cognitive deficits are made more manageable with support from a conversation partner [31], and the benefits of training caregivers of this population may extend to social behaviour.

There was no evidence that these associations were confounded by variations in age, ACE-III score, conversation location, or conversation partner. This suggests that the benefits of training interventions may not be limited by varying age or cognitive impairment of people with mild AD or the context in which learned support is provided. Evidence from the individual MSC-D functions of support revealed that while revealing competence for understanding, responding, and verification scores were positively associated with better social behaviour, acknowledging competence scores had no such association. This suggests that different functions of support have varying effects on social behaviour, and although we cannot confirm the absence of an association between acknowledging competence and social behaviour due to the limited power of our analyses, understanding that different functions may be more or less important than others could inform approaches to social behavioural support. More research is needed to further explore these relationships, guiding the development of targeted interventions aimed at promoting and sustaining social behaviour as the illness progresses.

Comparison of conversations with familiar and unfamiliar conversation partners revealed no difference in the social behaviour of the participants with AD. In the unadjusted models, the participation of people with AD was better with the unfamiliar researchers than the familiar conversation partners, but when we adjusted for the effect of support—the researchers scoring higher for support than the family/friend participants—this association was attenuated. Therefore, while we found preliminary evidence for a relationship between support from the conversation partner and the appropriateness of social behaviour in AD, there was little to no evidence that familiarity of the conversation partner influenced the social behaviour observed in our study. It may be that our choice of a neutral conversation topic influenced this finding; autobiographical memory theory would suggest that emotional memory is a powerful tool in the retrieval of social information [15], thus a less neutral topic may allow for the effect of familiarity on social behaviour to be observed. Future research should test whether conversations on more meaningful issues to the person with dementia would favour a familiar conversation partner.

These findings were unexpected and diverge from the principles of cognitive rehabilitation, which suggest that leveraging familiar associations can reduce cognitive load, enhancing performance on cognition-based tasks [24]. However, evidence indicates that social cognition operates separately from more general cognitive processes [32]. This distinction may account for the lack of transferability of the effect of reduced cognitive load on memory tasks [14] to tasks involving social cognitive processes. In line with this theoretical explanation of our findings, the social context network model, attributing social behavioural disturbances in bvFTD to impaired social perception of contextual cues [5], may offer a more suitable explanatory framework. Applying this model to the AD population is further supported by recent evidence that social perception impairment is comparable between bvFTD and AD [33]. Nevertheless, considering the limitations of the current study, further research is required to determine whether the observed lack of an association between familiarity of conversation partner and social behaviour in AD can be replicated in larger and more diverse samples.

### Limitations

While this study had significant methodological strengths, including the use of direct behavioural observation and independent ratings of social behaviour, there were also limitations. The sample size was relatively small, and the power in some analyses was limited. The reduced power was also related to our decision to use multilevel modelling to account for clustering of participants with AD [34]. Although there is evidence that two-level models can tolerate such sparse data [29], and our assumptions check revealed suitable clustering for multilevel modelling (see the Appendix B) [35], the random effects structure may be unstable. This could explain the non-significance of a relationship between the familiarity of the conversation partner and the appropriateness and participation in social behaviour. This study was also restricted in its external validity and may not generalise to other populations. For example, our sample was mostly White and relatively well educated; higher levels of education may support increased cognitive reserve, compensating for the effects of mild AD on communication. The MSC-D, MPC-D, and SOI have not been extensively used since their development, so there is little evidence for their validity. The SOI was developed for use with bvFTD populations and not validated in AD. However, our findings on the reliability of these scales support their use as adapted scales for use with dementia populations [11]. Furthermore, the method of using these measures to code conversations meant that the raters could not be blinded to the participant they were observing (i.e., participants with AD, their relatives, or researchers). Thus, observer bias may have influenced the coding of conversations due to expectations of social behaviour and support. Sequence effects may also confound the evidence reported, as we did not counterbalance the conversation order (i.e., all the participants with AD had a conversation first with their relative and second with a researcher). Another influential factor in the data collection process is the effect of being filmed on social behaviour. This effect is unknown, but the participants may have behaved less naturally due to being aware of being filmed [23].

## 5. Conclusions

The current study supports the utility of conversation as a valuable paradigm for observing and exploring the social behaviour of people with mild AD and its covariates. Our findings are preliminary and suggest that greater support from an unimpaired conversation partner is associated with more appropriate social behaviour of people with mild AD, indicating the potential impact for people with dementia of improving conversational support. This suggests the need for testing and implementing training programmes to improve conversational support targeted at informal carers and at professionals. Our study also highlights the importance of developing an understanding of social behavioural changes in dementia and strengthens the call for more research using longitudinal and interventional studies and larger and more diverse samples across varying subtypes and stages of the illness.

## Figures and Tables

**Table 1:** Participant demographic and clinical characteristics. N = 19 dyads.

		Participants with AD (n = 19)		Familiar Conversation Partners (n = 19)	
		n	Mean (SD) or %	n	Mean (SD) or %
Mean age		19	81 (6)	19	67 (15)
Gender					
	Female	11	58%	13	68%
	Male	8	42%	6	32%
Ethnicity					
	White	13	68%	14	74%
	Black, African, Caribbean, or Black British	1	5%	1	5%
	Mixed or Multiple Ethnicity	1	5%	3	16%
	Asian or Asian British	1	5%	0	0%
	Other—“British Jewish”	2	11%	1	5%
	Other—“Mediterranean British”	1	5%	0	0%
First language					
	English	15	79%	15	79%
	Other	4	21%	4	21%
Marital Status					
	Married or in a partnership	12	63%	15	79%
	Divorced, separated, widowed, or single	7	37%	4	21%
Highest level of education					
	Postgraduate degree-level	4	21%	10	53%
	Degree-level	7	37%	5	26%
	Secondary school	5	26%	3	16%
	Primary school	3	16%	1	5%
ACE-III score		19	71 (12)	-	-

Note. ACE-III = Addenbrooke’s Cognitive Examination III; AD = Alzheimer’s dementia; SD = standard deviation.

**Table 2:** Inter-rater reliability of scores on the SOI, MPC-D, and MSC-D between rater 1 and rater 2 or 3. N = 38 conversations.

Measures of Social Behaviour (MPC-D and SOI) and Support (MSC-D)		n = 28		n = 10		ICC	95% CI	Mean (SD) of Scores Used in Further Analysis
		Rater 1	Rater 2	Rater 1	Rater 3			
		Mean Score (SD)	Mean Score (SD)	Mean Score (SD)	Mean Score (SD)			
MSC-D	Acknowledging competence	3.6 (0.4)	3.6 (0.7)	3.6 (0.7)	3.5 (0.9)	0.86	0.73, 0.93	3.6 (0.6)
	Revealing competence: understanding	3.4 (0.8)	3.5 (0.8)	3.7 (0.7)	3.9 (0.3)	0.75	0.52, 0.87	3.5 (0.7)
	Revealing competence: responding	3.3 (0.9)	3.5 (0.8)	3.6 (0.8)	3.6 (1.0)	0.87	0.76, 0.93	3.5 (0.8)
	Revealing competence: verification	2.5 (0.8)	2.4 (0.8)	2.7 (0.9)	2.7 (0.9)	0.64	0.32, 0.82	2.5 (0.7)
	Total scores	12.9 (2.3)	12.9 (2.5)	13.6 (2.5)	13.7 (2.1)	0.90	0.81, 0.95	13.1 (2.2)
MPC-D	Interaction	2.9 (0.7)	2.8 (0.8)	3.8 (0.3)	3.8 (0.4)	0.82	0.66, 0.91	3.1 (0.7)
	Transaction	3.2 (0.7)	3.0 (0.9)	3.8 (0.6)	3.9 (0.2)	0.76	0.54, 0.88	3.3 (0.7)
	Total scores	6.1 (1.2)	5.8 (1.6)	7.6 (0.6)	7.7 (0.4)	0.86	0.74, 0.93	6.4 (1.4)
SOI	Verbal deficits	44.0 (3.9)	45.5 (3.8)	46.8 (2.3)	48.5 (1.7)	0.87	0.60, 0.94	45.5 (3.5)
	Nonverbal deficits	45.9 (4.7)	45.9 (6.8)	49.5 (0.7)	49.2 (1.6)	0.92	0.85, 0.96	46.8 (5.0)
	Total scores	89.9 (7.5)	91.4 (9.6)	96.3 (2.6)	97.7 (2.5)	0.92	0.85, 0.96	92.3 (7.7)

Note. All coefficients (ICC) are significant at *p* < 0.05; CI = confidence interval; ICC = intraclass correlation coefficient; MPC-D = Measure of Participation in Conversation for Dementia; MSC-D = Measure of Support in Conversation for Dementia; SD = standard deviation; SOI = Social Observation Inventory.

**Table 3:** Model estimates for the association between support from the conversation partner (MSC-D scores) and the social behaviour of people with AD (MPC-D and SOI scores). Clusters = 19.

		Social Behaviour of People with AD		
Multilevel Linear Regression Models		β	SE	95% CI
Model 1: MPC-D score				
MSC-D score (unadjusted)		0.27	0.06	0.16, 0.39
	Adjusted for age of participants with AD	0.27	0.06	0.16, 0.39
	Further adjusted for ACE-III score	0.28	0.06	0.17, 0.39
	Further adjusted for location of conversation	0.26	0.06	0.15, 0.38
	Further adjusted for conversation partner (fully adjusted)	0.29	0.07	0.14, 0.44
Model 2: SOI score				
MSC-D score (unadjusted)		1.33	0.28	0.78, 1.88
	Adjusted for age of participants with AD	1.30	0.28	0.74, 1.85
	Further adjusted for ACE-III score	1.30	0.28	0.74, 1.85
	Further adjusted for location of conversation	1.25	0.28	0.70, 1.81
	Further adjusted for conversation partner (fully adjusted)	1.59	0.37	0.87, 2.32

Note. All effect sizes (β) are significant at *p* < 0.001; ACE-III = Addenbrooke’s Cognitive Examination III; AD = Alzheimer’s dementia; CI = confidence interval; MPC-D = Measure of Participation in Conversation for Dementia; MSC-D = Measure of Support in Conversation for Dementia; SE = standard error; SOI = Social Observation Inventory.

**Table 4:** Model estimates for the association between individual MSC-D function scores and MPC-D and SOI scores. Clusters = 19.

	Social Behaviour of People with AD					
Multilevel Linear Regression Models	Unadjusted β	SE	95% CI	Fully Adjusted β	SE	95% CI
MPC-D score						
Acknowledging competence	0.81 **	0.25	0.32, 1.31	0.61	0.35	−0.08, 1.30
Revealing competence: Understanding	0.98 ***	0.23	0.53, 1.43	1.10 **	0.32	0.48, 1.73
Revealing competence: Responding	0.74 ***	0.17	0.41, 1.07	0.79 **	0.23	0.33, 1.25
Revealing competence: Verification	0.57 *	0.23	0.11, 1.03	0.57 **	0.20	0.17, 0.96
SOI score						
Acknowledging competence	3.13 *	1.33	0.52, 5.73	2.31	1.93	−1.46, 6.09
Revealing competence: Understanding	5.34 ***	1.02	3.35, 7.34	7.33 ***	1.39	4.60, 10.05
Revealing competence: Responding	3.57 ***	0.78	2.05, 5.10	4.94 ***	1.06	2.86, 7.03
Revealing competence: Verification	3.06 **	1.15	0.81, 5.32	2.78 *	1.08	0.67, 4.89

Note. Fully adjusted models are adjusted for the age of the participants with AD, ACE-III scores, location of conversation, and conversation partner. *p* < 0.05 *, *p* < 0.01 **, *p* < 0.001 ***; ACE-III = Addenbrooke’s Cognitive Examination III; AD = Alzheimer’s dementia; CI = confidence interval; MPC-D = Measure of Participation in Conversation for Dementia; MSC-D = Measure of Support in Conversation for Dementia; SOI = Social Observation Inventory.

**Table 5:** Model estimates for the effect of conversation partner on MPC-D and SOI score. Clusters = 19.

		Social Behaviour of People with AD		
Multilevel Linear Regression Models		β	SE	95% CI
Model 3: MPC-D score				
Conversation partner (familiar or unfamiliar) (unadjusted)		0.61 *	0.28	0.06, 1.15
	Adjusted for age of participants with AD	0.61 *	0.28	0.06, 1.15
	Further adjusted for ACE-III score	0.61 *	0.28	0.06, 1.15
	Further adjusted for location of conversation	0.61 *	0.28	0.06, 1.15
	Further adjusted for MSC-D score (fully adjusted)	−0.14	0.30	−0.73, 0.46
Model 4: SOI score				
Conversation partner (familiar or unfamiliar) (unadjusted)		2.18	1.40	−0.56, 4.93
	Adjusted for age of participants with AD	2.18	1.40	−0.56, 4.93
	Further adjusted for ACE-III score	2.18	1.40	−0.56, 4.93
	Further adjusted for location of conversation	2.18	1.40	−0.56, 4.93
	Further adjusted for MSC-D score (fully adjusted)	−1.93	1.41	−4.68, 0.83

Note. Reference group = familiar conversation partner; *p* < 0.05 *; ACE-III = Addenbrooke’s Cognitive Examination version 3; AD = Alzheimer’s dementia; CI = confidence interval; MPC-D = Measure of Participation in Conversation—Dementia; MSC-D = Measure of Support in Conversation—Dementia; SE = standard error; SOI = Social Observation Inventory.

## Data Availability

The video recording data presented in this study are not readily available due to confidentiality and ethical restrictions. Requests for other data should be directed to the corresponding author.

## Appendix A

### Appendix A.1. Instructions to Be Read by Researcher at Start of Task

Part 1: Video-recorded social interaction between participant and informant

Researcher (to dyad): ‘I would like you to speak to each other for about 5–10 min about the favourite foods of [the participant]. Talk about what the food is, what you like about it, how you prepare it or where you buy it, and times you remember eating it.

You should both speak as freely as you like but if you would like to be reminded of the task, then [the informant] can look at this prompt sheet.’

Part 2: Video-recorded interaction between participant and researcher

Research (to person with Alzheimer’s dementia): ‘Please tell me about your favourite food. Talk about what the food is, what you like about it, how you prepare it or where you buy it, and times you remember eating it.’

Prompts:

Tell me about a food you really enjoy

- What do you like about it?
- What does it taste like?
- How do you make it?
- OR: Where do you buy it from?
- Tell me about some times you remember eating it

- Tell me more about these times
- Do you eat it on particular occasions?

### Appendix A.2. Prompt Sheet for Family/Friend of Person with Dementia

Here are some things you might like to speak about:

Tell me about a food you really enjoy

- What do you like about it?
- What does it taste like?
- How do you make it?

OR: Where do you buy it from?

- Tell me about some times you remember eating it

- Tell me more about these times
- Do you eat it on particular occasions?

## Appendix B. Assumption Checks for Statistical Analyses

Histogram plots of the distribution of data for variables SOI, MPC-D, and MSC-D revealed negative skews for all three, informing non-parametric testing of differences between conversations with researchers and relatives.

Histogram and scatter plots of residuals revealed that the assumptions of normality and homoscedasticity were satisfactorily met at the individual and cluster levels for multilevel linear regression model 1, but not model 2. This suggests a non-constant variance in errors in model 2 (Table 3). Histogram and box plots of residuals revealed that the assumptions of normality were met at the cluster level and the assumptions of no extreme outliers were met at both the cluster and individual levels for model 3, but neither the assumption of normality nor no extreme outliers were met for model 4, suggesting non-constant variance in errors (Table 5). ICC estimates found >50% variance between clusters in all four models, supporting the decision to use multilevel modelling [35].

The linearity of the relationships explored in models 1 to 4 was checked by conducting multilevel linear regression models of the associations between the continuous explanatory and outcome variables and introducing quadratic terms for the explanatory variables as covariates. The assumptions were met for all except the relationship between the SOI score and MSC-D score, for which there was evidence for a curvilinear relationship (β = −0.29; 95% CI: −0.50 to 0.08; *p* = 0.007).

## Appendix C. Sensitivity Analyses

Considering our small sample size and evidence for non-constant variance in errors in models 2 and 4, we conducted bootstrapping of the reported coefficients for all four fully adjusted models (see Table 3 and Table 5). Here, we report robust standard errors (SEs) for all four fully adjusted models: model 1 SE = 0.07 (β = 0.29; 95% CI: 0.15, 0.43), model 2 SE = 0.45 (β = 1.59; 95% CI: 0.72, 2.47), model 3 SE = 0.32 (β = −0.14; 95% CI: −0.77, 0.49), and model 4 SE = 1.04 (β = −1.93; 95% CI: −3.97, 0.11).

To address the potential for a curvilinear relationship between the SOI and MSC-D scores, multilevel linear regression models 2 and 4 were repeated using quadratic terms for the MSC-D scores in the models and adjusting for heteroscedasticity in residuals (see Table A1). Our analyses revealed evidence for a relationship between the SOI score and MSC-D score: in the fully adjusted model, every 1-point higher MSC-D score was associated with an 8.37 higher (95% CI: 5.08 to 11.67) SOI score. There was no evidence for an association between the SOI score and familiarity of the conversation partner until the model was adjusted for heteroscedasticity in residuals; in the fully adjusted model, SOI scores were 1.81 lower (95% CI: −3.54 to −0.07) during conversations with unfamiliar conversation partners compared to familiar partners.

**Table A1:** Model estimates for the effect of MSC-D scores and conversation partner on MPC-D and SOI scores, adjusting for quadratic covariates and heteroscedasticity in residuals. Clusters = 19.

		Social Behaviour of People with AD	
Multilevel Linear Regression Models		β	95% CI
SOI scores			
MSC-D scores (unadjusted)		1.33 ***	0.78, 1.88
	Adjusted for MSC-D scores quadratic term	8.29 **	3.22, 13.37
	Further adjusted for heteroscedasticity in residuals	8.29 ***	4.07, 12.52
	Further adjusted for age of participants with AD, ACE-III scores, conversation location, and conversation partner (fully adjusted)	8.37 ***	5.08, 11.67
Conversation partner (familiar or unfamiliar) (unadjusted)		2.18	−0.56, 4.93
	Adjusted for MSC-D score quadratic term	−2.06	−4.36, 0.25
	Further adjusted for heteroscedasticity in residuals	−2.06 *	−3.98, −0.22
	Further adjusted for age of participants with AD, ACE-III scores, conversation location, and conversation partner (fully adjusted)	−1.81 *	−3.54, −0.07

Note. *p* < 0.05 *; *p* < 0.01 **; *p* < 0.001 ***; ACE-III = Addenbrooke’s Cognitive Examination III; AD = Alzheimer’s dementia; CI = confidence interval; MPC-D = Measure of Participation in Conversation for Dementia; MSC-D = Measure of Support in Conversation for Dementia; SOI = Social Observation Inventory.
